# Market risk, financial distress and firm performance in Vietnam

**DOI:** 10.1371/journal.pone.0288621

**Published:** 2023-07-19

**Authors:** Duc Hong Vo

**Affiliations:** Research Centre in Business, Economics & Resources, Ho Chi Minh City Open University, Ho Chi Minh City, Vietnam; Bucharest University of Economic Studies: Academia de Studii Economice din Bucuresti, ROMANIA

## Abstract

In 2021, when the Covid-19 pandemic had a severe impact on the economy, a significant number of enterprises in Vietnam temporarily suspended doing business. Previous studies have focused on either model for predicting bankruptcy and financial distress or measuring market risk during extreme events. The effects of market risk and financial distress on a firm’s performance have largely been ignored in the literature, particularly in Vietnam. This study examines the effects of market risk, measured using the conditional value-at-risk technique and financial distress proxied by the interest coverage ratio (ICR) on firm performance for 500 nonfinancial listed firms in Vietnam from 2012 to 2021. We also estimate the optimal ICR for Vietnam’s listed firms. Two estimation techniques are used: dynamic panel models (two-step difference–and system–generalized method of moments) and panel threshold regression. We find that increased market risk reduces firm performance. However, a higher ICR (lower financial distress) also improves a firm’s performance. With increased market risk, the financial performance of firms with a high ICR deteriorates significantly.

## 1. Introduction

The COVID-19 pandemic has changed the world. A significant number of firms across countries have struggled to survive during the pandemic. These firms have faced financial distress, generally referred to as a situation in which a firm struggles to fulfill its short-term financial obligations [1, 2]. In particular, financial distress emerges when a firm cannot generate sufficient earnings to cover its financial obligations. An extended period of financial distress leads to bankruptcy for firms. Since the COVID-19 pandemic emerged in December 2019, firms in many countries have experienced distress. Vietnam, despite its impressive economic growth, is no exception.

**Fig 1:**
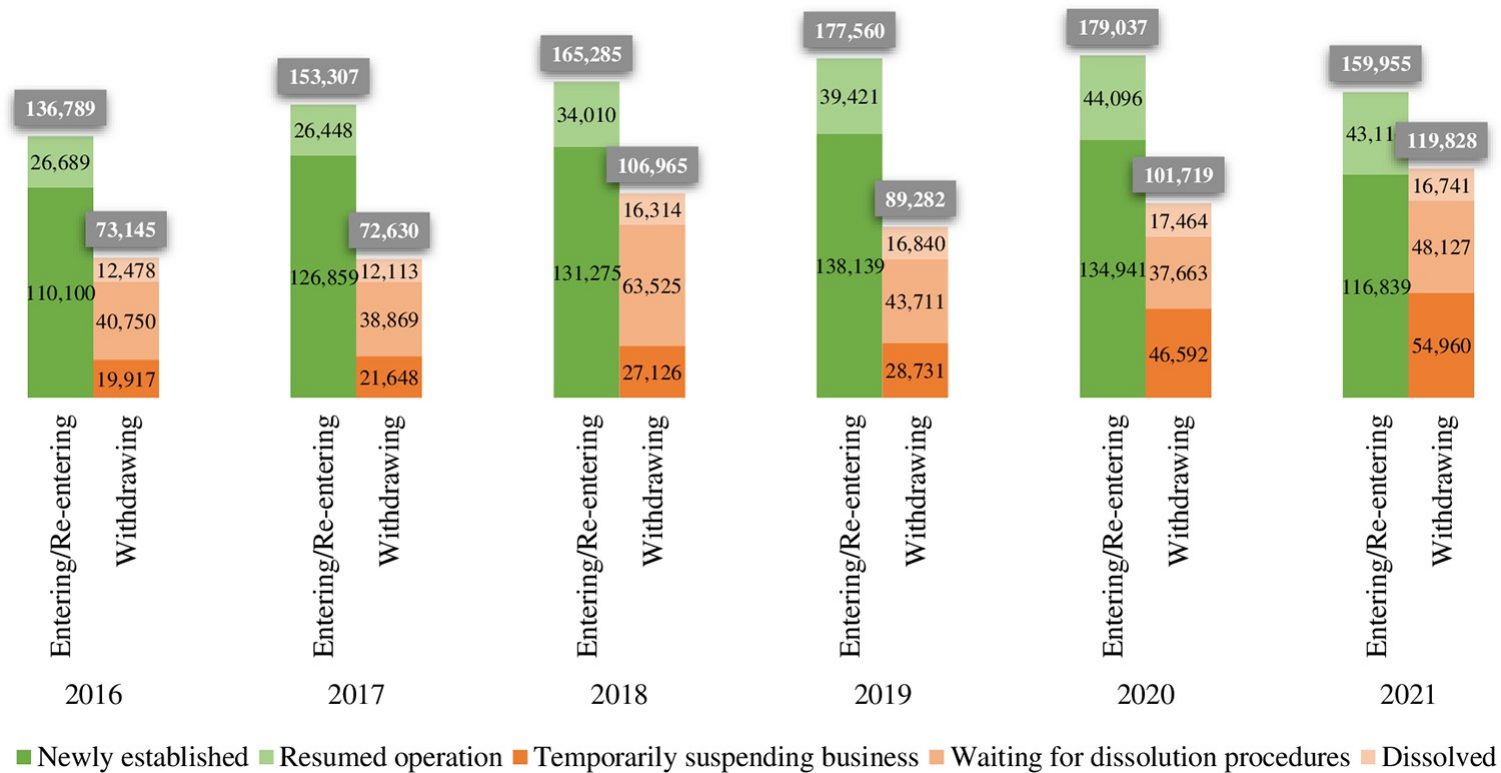
Business registration in Vietnam from 2016 to 2021. Source: General Statistics Office [3–8].

The development of business activities is illustrated in Fig 1. During the period 2016–2019, the number of enterprises in Vietnam that entered and re-entered the market gradually grew, and the number of enterprises that exited the market was relatively stable, except in 2018, when a record-high number of enterprises filed for bankruptcy proceedings. However, the Covid-19 pandemic during the period 2020–2021 caused considerable disruption for enterprises in Vietnam. As a result, the number of enterprises that entered and re-entered the market grew slightly in 2020 and plunged in 2021. In addition, the number of enterprises withdrawing from the market increased significantly. In particular, far fewer enterprises were newly established in 2020 than in 2019. However, thanks to a record increase in enterprises that resumed operations, the number of enterprises entering and re-entering the market in 2020 still increased over the number in 2019. In 2021, however, when the number of enterprises newly established and resuming operations fell significantly, this pattern collapsed. In addition, the significant increase in the number of enterprises leaving the market was mainly due to the large number of enterprises that temporarily suspended doing business.

The enterprises that temporarily suspended activity experienced financial distress, which, if prolonged, will lead to dissolution or bankruptcy. Thus, diagnosing financial distress by examining firm performance is critical, especially during extreme events in which the market risk is intensified. However, previous studies focus only on either building models to predict bankruptcy and financial distress [9–13] or measuring market risk during extreme events [14–18]. No studies to date have combined these two separate effects, market risk and financial distress, on firm performance. As such, this study examines the impact of market risk and financial risk on firm performance to fill a gap in the existing literature. This study examines the effects of market risk, measured using the value-at-risk (VaR) technique and financial distress using the interest coverage ratio (ICR), on the financial performance of 500 nonfinancial listed firms in Vietnam from 2012 to 2021.

Over the past two decades, Vietnam has achieved among the world’s highest economic rates of growth, becoming one of the 41 largest economies. The economy ranks fifth among the members of the Association of Southeast Asian Nations (ASEAN) [19] and is among the 16 most successful emerging economies globally [20]. However, in the third quarter of 2021, Vietnam experienced its first-ever negative rate of growth in the gross domestic product (GDP) since 2000 [8]. Although Vietnam ended the year 2021 with a positive GDP annual growth rate of 2.58 percent, this growth rate was significantly below the rate attained in the prior 22 years [21]. However, in 2022, Vietnam again achieved economic growth of 8.02 percent, the fastest pace since 1997. This rapid growth enabled significant positive and effective development of business activities, which comprise over 60 percent of Vietnam’s GDP. As such, the failure of business operations in Vietnam has attracted great attention from policy makers, particularly in view of its ambition to become a middle-income country by 2040. Understanding the effects of the market risks and financial distress, particularly their combined impacts, on a firm’s performance in Vietnam is critical for formulating and implementing economic policies that support successful operations by Vietnamese firms. Lessons from Vietnam’s experience, particularly during periods of instability such as the ongoing pandemic, the war in Ukraine, and many global geopolitical risks, would be valuable for other emerging markets.

The objective of this study is to determine: the extent of market risk’s impact on firm performance, the effects of financial distress on firm performance, and the combined effects of market risk and financial distress on firm performance.

The study makes two contributions to the existing literature. First, this paper is one of the first to investigate the effects of market risk on firm’s performance, particularly for Vietnam. Second, this study simultaneously examines the combined effects of both market risk and financial risk on firm performance, in particular for emerging markets, such as Vietnam. Our empirical findings have significant implications for the Vietnamese government regarding the implementation of relevant regulations and policies to support firms during and after major market risk events and practitioners regarding investment analysis.

Following this introduction, the remainder of this paper is structured as follows. Section 2 discusses and synthesizes the related literature and presents the research hypotheses. Section 3 presents the data, sample, and research methodology. Section 4 reports and discusses the empirical results. Finally, the concluding remarks and implications are discussed in Section 5.

## 2. Literature review and research hypotheses

### 2.1 Theoretical background in market risk and financial distress

Market risk is inevitable, and it systemically affects the entire economy in which firms operate daily. The exposure to market risk is commonly in the form of fluctuations in interest rates, foreign exchange rates, share prices, and commodity prices [22]. Among these forms of market risk, share price fluctuations have a large effect on market asset value (Allen & Powell, 2012), a market-based measurement of firm performance [23]. Therefore, capturing the effect of market risk on firm performance through equity price fluctuations is practical work, especially during extreme events [24]. In addition, financial distress is generally considered a condition in which firms cannot meet their short-term financial obligations [1, 2, 24]. This type of distress can cause firms to go bankrupt. The theoretical basis of financial distress mainly refers to liquidity and profitability, such as liquidity and profitability theory, the balance-sheet decomposition measure, and liquidity risk theory.

Most early studies on financial distress focus extensively on the firm-specific effects of various factors. Specifically, financial ratios are often used to detect the financial health of firms. Significant financial ratios include profitability and the leverage ratio [9–13], the liquidity ratio [25–28], cash-flow ratios [29, 30], and the ICR [31–34].

Our literature review also confirms that recent papers have examined factors that can affect the probability of financial distress at firms. These factors include leverage, operating leverage, ownership structure, earnings management, board gender diversity, and corporate social responsibility. Specifically, the important role of financial leverage, determining the level of borrowing by firms, is crucial in understanding and explaining financial distress risk. Financial leverage is generally seen as affecting default risk, which is more significant for small and medium-size enterprises than for large enterprises [35]. Firms with higher financial leverage are generally associated with higher financial distress, particularly during a significant market event, such as the pandemic. Moreover, operating leverage can also increase the risk of financial distress and default [36]. In addition, firms can manage and enhance real earnings management to reduce financial distress by increasing the proportion of women on the board of directors or increasing participation in corporate social responsibility activities [37–39].

The link between market risk, financial distress, and firm performance can be established as follows. The market risks are embedded in the share prices, which effectively affects the market value of a firm’s equity. As a result, firm value effectively decreases. In addition, firm value has been found to affect firm performance [24, 40]. Large firms (i.e., firms with high market capitalization and total firm value) are found to have higher firm performance due to their market power in pricing and competition. In addition, the fluctuations in share prices cause a decline in the value of equity and total firm value. A reduction in firm value makes it more difficult for firms to borrow to finance their business operations. As such, financial distress deteriorates further because of two effects: first, firms now find it more challenging to access additional funding from financial institutions and the markets, and second, firms currently face financial distress. As such, they cannot meet their short-term financial obligations because of a lack of internal funding sources. On balance, the effects of financial distress will deteriorate further at a given level of the market risks arising from significant fluctuations in share prices.

### 2.2 Market risk and firm performance

The extreme value theory offers a comprehensive basis for constructing market risk estimators under extreme scenarios [41] in which value-at-risk (VaR) and conditional value-at-risk (CVaR) are among the most typical estimators of market risk. However, VaR is inferior to CVaR, and the model cannot capture the scenarios in which the VaR estimates are exceeded [42, 43]. In other words, VaR may understate the level of risk because the estimator ignores all returns less than the estimated VaR at a given confidence level. Meanwhile, CVaR can overcome this limitation by considering the expected losses exceeding the estimated VaR at the same confidence level [44]. CVaR offers an average expected loss, instead of a wide range provided by VaR, which is difficult to consider. A market risk measure, such as CVaR, is generally considered a standard tool for detecting early warning signals of distress in the real economy [45]. Thus, CVaR can be used to proxy for market risk that affects firm performance.

Our literature review indicates that the current literature uses only the CVaR approach to compare market risk across different extreme events, including the global financial crisis [15, 17, 38] and the Covid-19 pandemic [14, 16] or estimate the market risk in the commodity market [40, 46] and foreign exchange market [47]. The main findings in these studies suggest that market risk soars during the peak of extreme events. However, none of the previous studies has applied CVaR to the study of firm performance. Thus, we pioneer using the CVaR approach to measure market risk, which is then used to investigate the effect of market risk on firm performance in Vietnam. We test the following hypothesis.

**Hypothesis 1.** An increase in market risk is associated with a decline in a firm’s performance.

### 2.3 The role of the ICR in financial distress and its effect on firms’ performance

Among such financial ratios, the ICR, an indicator of the firm’s capacity to make its interest payments relying on the internal cash flow, appears to be particularly attractive. The reason is that ICR can be used to evaluate the probability of default for firms without credit ratings [13, 48], which is suitable for emerging markets with immature credit ratings. For instance, the prediction model measured by ICR can signal financial distress one year ahead for most firms in the ASEAN region [24]. In addition, ICR can act as a critical financial distress indicator with serious implications for monetary policy design [49, 50].

**Hypothesis 2.** An increase in ICR is associated with an improvement in firm performance.

The ICR is estimated as the ratio of earnings before interest and taxes to interest expenses. Asquith [32] consider a firm financially distressed if its ICR is below 0.8, whereas Andrade and Kaplan [31] and Fan [34] put firms into a distressed zone when the ICR is less than 1. Thus, a high ICR indicates a low level of financial distress for firms. In other words, firms with high ICR levels are expected to have better performance. This study examines the potential effect of the ICR as a determinant of firm performance and as a shield to protect firms from the detrimental impact of market risk. Based on these observations, we form our second and third hypotheses.

**Hypothesis 3.** A high ICR (low financial distress) mitigates the detrimental impact of market risk on firm performance.

Our literature review indicates that the effects of market risks on firm performance have largely been underexamined in the existing literature. The market risks matter for a firm’s performance. In addition, the combined effects of market risk and financial distress risk have also been largely ignored in previous empirical analyses. At a given level of market risk, the effects of financial distress on a firm’s performance may have further deteriorated. These rationales motivate us to conduct this analysis.

## 3. Data and research methodology

### 3.1 Data and sampling

We use data on 500 nonfinancial listed firms in Vietnam to estimate the effects of market risk and financial risk on firm performance. Fiscal year-end financial data on firms are collected from Thomson Reuters from 2012 to 2021. These financial data include total assets, total debt, total equity, current assets, current liabilities, net income, earnings before interest and taxes (EBIT), interest expense, and the firm’s year of establishment. In addition, we collect the firms’ daily historical stock prices from January 2, 2012, to December 31, 2021 from the Ho Chi Minh City Stock Exchange (HOSE) and Hanoi Stock Exchange (HNX) to calculate yearly market risk using the CVaR method.

The return on assets (*ROA*) and return on equity (*ROE*) are calculated from the ratio of net income to total assets and net income to total equity, respectively. The interest coverage ratio (*ICR*) is calculated from the ratio of EBIT to interest expense. Any ICR value that exceeds the median in the sample is considered high (*HighICR*). Firm age (*AGE*) is the firm’s years of establishment. Firm size (*SIZE*) is measured by the firm’s total assets. The degree of financial leverage (*DFL*) is the ratio of EBIT to the difference between EBIT and interest expense. The leverage ratio (*LEV*) is the ratio of total debt to total assets. The current ratio (*CUR*) is the ratio of current assets to current liabilities. The measurement of market risk using the CVaR (*CVaR*) is elaborated in the next section.

*ROA* and *ROE* are used as proxies for firm performance [51, 52]. For explanatory variables, we use *CVaR* and *ICR* to proxy for market risk [16, 53] and financial distress risk [13, 24]. We use *HighICR* as a dummy variable to interact with market risk. We use *AGE*, *SIZE*, *DFL*, *LEV*, and *CUR* to control for other factors that might affect firm performance [54–57]. Table 1 summarizes the descriptions, measurements, and sources of all the variables used in the study.

**Table 1:** Description, measurement, and source of variables.

Variable	Description	Measurement	Source
**Response variables**			
*ROA*	Return on assets	Net income/Total assets	Thomson Reuters
*ROE*	Return on equity	Net income/Equity	
**Explanatory variables**			
*CVaR*	Conditional value-at-risk	*CVaR* = *E*(*r*|*r*≥*VaR*) where *r* denotes stock returns	Author calculation
*ICR*	Interest coverage ratio	Earnings before interest and taxes (EBIT)/Interest expense	Thomson Reuters
*HighICR*	High interest coverage ratio	A dummy variable equals one if ICR exceeds the median in the sample and zero otherwise.	Author calculations
**Control variables**			
*AGE*	Firm age	Number of years since the firm’s establishment	Thomson Reuters
*SIZE*	Firm size	Total assets	
*DFL*	Degree of financial leverage	EBIT/(EBIT–Interest expense)	
*LEV*	Leverage ratio	Total debt/Total assets	
*CUR*	Current ratio	Current assets/Current liabilities	

### 3.2 Model specification

We apply various dynamic panel models to comprehensively examine the effects of market risk and financial distress on firm performance. The dynamic panel models can avoid the endogeneity problem and capture the dynamic effects, which is typical in the corporate finance context. We add the lagged dependent variable (*Firm performance*_*i*,*t*−1_) to consider the dynamic effects of market risk and financial distress on firm performance. These dynamic panel models are expressed in Eqs (1)–(4) as follows:

$$Firm\;performance_{i,t}=\gamma_{0}+\gamma_{1}Firm\;performance_{i,t-1}+\gamma_{2}{CVaR}_{i,t}+\gamma_{3}{ICR}_{i,t}+\gamma_{4}Performance\;control_{i,t}+Year\;dummies+\varepsilon_{i,t}$$ (1)

$$Firm\;performance_{i,t}=\theta_{0}+\theta_{1}Firm\;performance_{i,t-1}+\theta_{2}{CVaR}_{i,t}+\theta_{3}{ICR}_{i,t}+\theta_{4}High{ICR}_{i,t}+\theta_{5}{HighICR\times CVaR}_{i,t}+\theta_{6}{Performance\;control}_{i,t}+{Year\;dummies}_{i,t}+\varepsilon_{i,t}$$ (2)

$$Firm\;performance_{i,t}=\lambda_{0}+\lambda_{1}Firm\;performance_{i,t-1}+\lambda_{2}{CVaR}_{i,t}+\lambda_{3}{ICR}_{i,t}+\lambda_{4}{ICR}_{i,t}^{2}+\lambda_{5}Performance\;control_{i,t}+Year\;dummies+\varepsilon_{i,t}$$ (3)

$$Firm\;performance_{i,t}=\varphi_{0}+\varphi_{1}Firm\;performance_{i,t-1}+\varphi_{2}{CVaR}_{i,t}+\varphi_{3}{ICR}_{i,t}+\varphi_{4}{ICR}_{i,t}^{2}+\varphi_{5}{HighICR}_{i,t}+\varphi_{6}{HighICR\times CVaR}_{i,t}+\varphi_{7}Performance\;control_{i,t}+Year\;dummies+\varepsilon_{i,t}$$ (4)

Table 2 lists the descriptive statistics of the variables used in our study. Our sample includes 500 nonfinancial firms listed in Vietnam for 10 years. The mean *ROA* is 0.056, lower than that of *ROE*, 0.102. The standard deviation of *ROA* and *ROE* are 0.083 and 0.272, respectively. The mean and standard deviation of *CVaR* are 0.06 and 0.019, respectively. *ICR* shows a large standard deviation of 34.009, with a minimum of -1.916 and a maximum of 239.027, meaning that the distribution is dispersed and skewed. The mean *AGE* and *SIZE* are 9.162 and 139.3 million, respectively, signifying that our sample comprises mature and large firms. These firms have, on average, varying degrees of financial leverage (*DFL*), with mean and standard deviation of 3.68 and 144.747, respectively. Also, the mean and standard deviation of *LEV* are 0.503 and 0.224, respectively, which implies that Vietnamese listed firms have different capital structures.

**Table 2:** Descriptive statistics.

Variables	N	Mean	SD	Min	Max
*ROA*	5,000	0.056	0.083	-1.59	0.78
*ROE*	5,000	0.102	0.27	-7.72	5.28
*CVaR*	4,953	0.06	0.019	0	0.16
*ICR*	4,790	18.2	34	-1.9	239.0
*HighICR*	4,790	0.5	0.5	0	1
*AGE*	4,993	9.162	6.337	0	61
*SIZE (‘000)*	5,000	139,300	658,100	466	18,720,000
*DFL*	4,959	3.68	145	-555	10,001
*LEV*	5,000	0.50	0.22	0.002	1.21
*CUR*	5,000	2.58	6.19	0	243

Table 3 illustrates the pairwise correlation coefficients among the variables in our study. No variables exhibit a significant collinearity problem. Our proxy for market risk–*CVaR* and firm performance–*ROA*, *ROE* have a negative relationship at the 1 percent level. In contrast, the correlation between the financial distress proxy *ICR* and firm performance is positive and significant at the 1 percent level. For the remaining variables, which are control variables, the correlations are below 0.80, thus the absence of multicollinearity in our analysis.

**Table 3:** The correlation matrix.

Variables	(1)	(2)	(3)	(4)	(5)	(6)	(7)	(8)	(9)
(1) *ROA*	1								
(2) *ROE*	0.551***	1							
(3) *CVaR*	-0.282***	-0.163***	1						
(4) *ICR*	0.342***	0.134***	-0.144***	1					
(5) ln*AGE*	0.0118	0.031*	-0.047**	-0.009	1				
(6) ln*SIZE*	-0.002	0.061***	-0.290***	-0.156***	0.167***	1			
(7) *DFL*	-0.011	-0.0034	0.014	-0.011	0.003	0.007	1		
(8) *LEV*	-0.343***	-0.074***	0.091***	-0.377***	0.001	0.348***	0.026	1	
(9) *CUR*	0.131***	0.017	0.002	0.214***	0.010	-0.161***	-0.007	-0.442***	1

Table 4 presents the results of the unit-root tests at the level and first difference using the augmented Dickey-Fuller (ADF) and Phillips-Perron (PP) tests. The null hypothesis of these two tests assumes a unit root at the level. The results indicate the rejection of the null hypothesis at the 1 percent significance level for all variables at both the level and first difference.

**Table 4:** Results of the unit-root tests.

Variables	Augmented Dickey-Fuller		Phillips-Perron	
	Fisher Chi-Square (ADF)		Fisher Chi-Square, (PP)	
	H_0_: I(0)	H_0_: I(1)	H_0_: I(0)	H_0_: I(1)
Firm Performance				
*ROA*	31.108***	23.109***	31.108***	32.19***
*ROE*	35.556***	31.600***	35.556***	37.97***
Market risk				
*CVaR*	17.443***	10.266***	17.443***	20.72***
Financial distress				
*ICR*	45.073***	25.015***	45.073***	46.27***
Control variables				
ln*AGE*	782.82***	781.02***	782.82***	782.82***
ln*SIZE*	5.33***	17.93***	5.33***	7.06***
*DFL*	117.4***	80.65***	117.4***	120.03***
*LEV*	6.132***	15.62***	6.13***	7.31***
*CUR*	19.005***	18.87***	19.005***	20.6***

*Note*: ADF: adjusted Dickey-Fuller (1981); PP: Phillips-Perron (1988). The null hypothesis H_0_: I(0) assumes a unit-root process at the level, whereas H_0_: I(1) assumes a unit root at the first difference. *** *p* < 0.01, ** *p* < 0.05, *** *p* < 0.1.

## 4. Empirical results

We follow a panel data approach to investigate the impact of market risk and financial distress on firm performance by Vietnamese listed firms. We include time dummies to control for any year-fixed effects that may affect our estimated results. Tables 5 and 6 present the empirical results from the dynamic models, which use two-step difference- and system-GMM estimators. Table 7 presents the empirical results using Hansen’s panel threshold estimation.

**Table 5:** The effects of market risk and financial distress on firm performance.

	Basic models				Basic models with interaction			
	Difference-GMM		System-GMM		Difference-GMM		System-GMM	
	(9)	(10)	(11)	(12)	(13)	(14)	(15)	(16)
Variables	*ROA*	*ROE*	*ROA*	*ROE*	*ROA*	*ROE*	*ROA*	*ROE*
*ROA*_t-1_	0.224***	N/A	0.213***	N/A	0.199***	N/A	0.182***	N/A
	(0.0414)		(0.0394)		(0.0405)		(0.0384)	
*ROE*_t-1_	N/A	-0.0157	N/A	-0.0263	N/A	-0.0249	N/A	-0.0381
		(0.0706)		(0.0691)		(0.0715)		(0.0693)
*CVaR*	-0.0491	-0.376*	-0.600***	-1.527***	0.00511	-0.365	-0.293***	-1.102***
	(0.0658)	(0.223)	(0.104)	(0.251)	(0.0794)	(0.279)	(0.0804)	(0.276)
*ICR*	0.00024***	0.00042***	0.00038***	0.00083***	0.00018***	0.00025***	0.0002***	0.00032***
	(0.00004)	(0.00008)	(0.00007)	(0.00015)	(0.00004)	(0.00007)	(0.00006)	(0.00012)
*HighICR*	N/A	N/A	N/A	N/A	0.0316***	0.0644***	0.0647***	0.122***
					(0.00694)	(0.0177)	(0.00921)	(0.0208)
*HighICR* * *CVaR*	N/A	N/A	N/A	N/A	-0.114	0.0265	-0.440***	-0.245
					(0.110)	(0.288)	(0.146)	(0.329)
*lnAGE*	-0.00700	-0.0324	0.00197	0.0133	-0.00492	-0.0242	0.00108	0.0108
	(0.00824)	(0.0206)	(0.00366)	(0.0111)	(0.00779)	(0.0196)	(0.00326)	(0.00955)
*lnSIZE*	0.0194***	0.0679***	0.00243**	0.00888***	0.0200***	0.0694***	0.00246**	0.0101***
	(0.00371)	(0.0191)	(0.00116)	(0.00326)	(0.00360)	(0.0190)	(0.00103)	(0.00282)
*DFL*	0.00001	0.00002	-0.000005	-0.00006	0.000001	0.00002	0.000005	-0.00003
	(0.00001)	(0.00009)	(0.00002)	(0.00007)	(0.00001)	(0.00009)	(0.00001)	(0.00006)
*LEV*	-0.132***	-0.203***	-0.0774***	-0.0125	-0.120***	-0.170**	-0.0495***	0.0698**
	(0.0177)	(0.0705)	(0.0108)	(0.0294)	(0.0176)	(0.0698)	(0.0105)	(0.0282)
*CUR*	0.00071	-0.00001	-0.00039	-0.00078	0.00077	0.00007	-0.00031	-0.00062
	(0.00108)	(0.00174)	(0.00086)	(0.00165)	(0.00106)	(0.00174)	(0.00085)	(0.00156)
Constant	−	−	0.0636***	0.0117	−	−	0.0179	-0.114**
			(0.0240)	(0.0585)			(0.0205)	(0.0519)
Year control	YES	YES	YES	YES	YES	YES	YES	YES
N	3763	3763	4242	4242	3763	3763	4242	4242
*p*-value AR(2)	0.282	0.368	0.355	0.372	0.258	0.366	0.333	0.366
*p*-value Hansen	0.577	0.349	0.702	0.376	0.595	0.415	0.688	0.492

Standard errors are in parentheses

*** p < 0.01

** p < 0.05

* p < 0.1. N/A indicates the variables excluded in the model.

**Table 6:** The quadratic relationship between ICR and firm performance using GMM estimators.

	Quadratic models				Quadratic models with interaction			
	Difference-GMM		System-GMM		Difference-GMM		System-GMM	
	(1)	(2)	(3)	(4)	(5)	(6)	(7)	(8)
Variables	*ROA*	*ROE*	*ROA*	*ROE*	*ROA*	*ROE*	*ROA*	*ROE*
*ROA*_t-1_	0.214***	N/A	0.203***	N/A	0.196***	N/A	0.181***	N/A
	(0.0392)		(0.0372)		(0.0393)		(0.0374)	
*ROE*_t-1_	N/A	-0.0181	N/A	-0.0292	N/A	-0.0255	N/A	-0.0383
		(0.0709)		(0.0702)		(0.0716)		(0.0697)
*CVaR*	-0.0500	-0.361	-0.560***	-1.421***	0.00349	-0.362	-0.287***	-1.091***
	(0.0645)	(0.221)	(0.0966)	(0.239)	(0.0791)	(0.279)	(0.0789)	(0.274)
*ICR*	0.0007***	0.00144***	0.00112***	0.00267***	0.00044***	0.0007***	0.00055***	0.00096***
	(0.0001)	(0.00027)	(0.00015)	(0.00034)	(0.00010)	(0.00024)	(0.00015)	(0.00030)
*ICR* ^2^	-0.000003***	-0.000006***	-0.000005***	-0.00001***	-0.000001***	-0.000002**	-0.000002**	-0.000004**
	(0.0000006)	(0.000001)	(0.0000009)	(0.000002)	(0.0000005)	(0.000001)	(0.0000009)	(0.000002)
*HighICR*	N/A	N/A	N/A	N/A	0.0294***	0.0605***	0.0599***	0.113***
					(0.0068)	(0.0176)	(0.00894)	(0.0206)
HighICR*CVaR	N/A	N/A	N/A	N/A	-0.112	0.0298	-0.438***	-0.249
					(0.110)	(0.288)	(0.145)	(0.327)
ln*AGE*	-0.0067	-0.0327	0.00225	0.0143	-0.00493	-0.0247	0.00137	0.0114
	(0.00815)	(0.0204)	(0.00345)	(0.0104)	(0.00778)	(0.0196)	(0.00321)	(0.00948)
ln*SIZE*	0.0197***	0.0676***	0.00283**	0.00992***	0.0202***	0.0692***	0.00260**	0.0103***
	(0.00363)	(0.0192)	(0.00112)	(0.00310)	(0.00357)	(0.0190)	(0.00102)	(0.00281)
*DFL*	0.000008	0.000018	-0.000002	-0.00005	0.000001	0.00001	0.000005	-0.00003
	(0.00001)	(0.00009)	(0.00002)	(0.00007)	(0.00001)	(0.00009)	(0.00001)	(0.00006)
*LEV*	-0.127***	-0.187***	-0.0665***	0.0172	-0.119***	-0.165**	-0.0479***	0.0729***
	(0.0176)	(0.0704)	(0.0103)	(0.0281)	(0.0176)	(0.0695)	(0.0104)	(0.0280)
*CUR*	0.00072	0.00001	-0.0005	-0.00101	0.00077	0.00007	-0.00036	-0.0007
	(0.00108)	(0.00175)	(0.00083)	(0.00157)	(0.00106)	(0.00174)	(0.00084)	(0.00155)
Constant	−	−	0.0462**	-0.0402	−	−	0.0155	-0.121**
			(0.023)	(0.0572)			(0.0208)	(0.053)
Year control	YES	YES	YES	YES	YES	YES	YES	YES
N	3763	3763	4242	4242	3763	3763	4242	4242
*p*-value AR(2)	0.304	0.371	0.425	0.377	0.273	0.367	0.365	0.368
*p*-value Hansen	0.596	0.338	0.643	0.357	0.605	0.408	0.666	0.478

Standard errors are in parentheses

*** p < 0.01

** p < 0.05

* p < 0.1. N/A indicates the variables excluded in the model.

**Table 7:** The optimal values of ICR using different estimators.

Estimators	ROA	ROE
Difference-GMM	117	120
System-GMM	112	134

### 4.1 The effects of market risk and financial distress on firm performance using dynamic panel models: Difference- and system-GMM estimators

An estimation problem arises in the presence of endogeneity, which is plausible in the corporate finance context. Specifically, firm performance proxied by ROA, ROE, and a financial distress indicator proxied by ICR may be simultaneously affected by certain omitted variables (e.g., revenue or the cost of goods sold). These omitted variables are incorporated into the error term, causing some correlation between the explanatory variable (ICR) and the error term. This explanatory variable-error term correlation, as a common source of endogeneity, violates the exogeneity assumption of the Gauss-Markov theory. These violations cause the estimated results from the conventional static panel models to be inconsistent and biased. Furthermore, the current level of ROA and ROE may be determined by their past values [58, 59] for which the specification of the static models is inadequate. Thus, the prevalent approach used to address these problems is the adoption of dynamic panel GMM estimators that offer valid and effective instruments.

We also use specification tests, including the AR(2) and Hansen overidentification tests, to assess the appropriateness of the GMM estimators. AR(2) is used to check for the absence of second-order autocorrelation in the first-difference error term, whereas the Hansen overidentification is used to check the overall validity of the choice of instruments. In general, the GMM estimators are consistent in the absence of second-order autocorrelation in the first-difference error term and when the Hansen test yields insignificant statistics.

Table 5 presents the effects of market risk and financial distress on firm performance using the two-step difference- and system-GMM estimators. We first look at the statistics from the AR(2) and Hansen overidentification tests. Both difference- and system-GMM estimators show no second-order autocorrelation in the first-difference error term and are appropriate instruments. We conclude that both GMM estimators are consistent, as indicated by the results in Table 5.

Based on the estimated results, both estimators’ coefficients for *ROA*_*t-1*_ and *ROE*_*t-1*_ are positive and significant. These results confirm that current firm performance is affected by prior performance. The coefficients for *CVaR* are negative but significant only with system-GMM, verifying the detrimental effects of market risk on firm performance. Meanwhile, both estimators’ coefficients for *ICR* are positive and significant, further confirming that increasing ICR improves firm performance. The coefficients for HighICR*CVaR with system-GMM are negative but only significant for ROA, implying that the performance of firms with a high ICR is damaged more with an increase in market risk. In other words, market risk deteriorates the financial performance of firms with a high ICR more significantly than those with a low ICR. This finding contradicts H3.

Table 6 presents the nonlinear (quadratic) relationship between financial distress (proxied by the ICR) and firm performance, using the two-step difference- and system-GMM estimators. The estimated coefficients of the quadratic term, *ICR*^2^, are negative and significant. The results suggest that firm performance improves up to the optimal level of the ICR.

The optimal ICRs obtained with different estimators, including difference- and system-GMM, are presented in Table 7. Across different proxies for firm performance using ROA and ROE, our findings confirm that the optimal value of ICR, the ratio between EBIT and interest expenses, varies between 112 and 134. These optimal values are derived with the system-GMM estimator.

### 4.2 Robustness analysis using Hansen (1999)’s panel threshold regression

This section discusses the empirical results of the market risk and financial distress effects on firm performance in Vietnam using the panel threshold regression by Hansen [60]. Table 8 presents the empirical results of this analysis, which confirm the structural changes in the relationship between ICR and firm performance. We find that both pre-threshold and at-threshold ICR positively affect firm performance, meaning that a higher ICR is associated with better firm performance. However, the estimated coefficient of ICR is smaller at the threshold than before the threshold. This finding means that firm performance increases more slowly when ICR reaches 38.4 (for ROA) and 22.2 (for ROE). As such, these findings imply that a higher ICR might slow down improvement in firm performance after a certain threshold is reached.

**Table 8:** The quadratic relationship between ICR and firm performance using a panel threshold regression model.

	ROA	ROE
*CVaR*	-0.184**	-1.050***
	(0.075)	(0.352)
*lnAGE*	-0.016***	-0.041***
	(0.002)	(0.01)
*lnSIZE*	0.028***	0.077***
	(0.002)	(0.011)
*DFL*	0.00002	-0.00005
	(0.0001)	(0.0002)
*LEV*	-0.161***	-0.246***
	(0.01)	(0.046)
*CUR*	0.001***	0.0003
	(0.0003)	(0.002)
***Pre-threshold***		
*ICR*	0.005***	0.017***
	(0.0004)	(0.003)
*ICR* ^*2*^	-0.0001***	-0.0007***
	(0.00001)	(0.0002)
***At threshold***	***ICR = 38*.*4***	***ICR = 22*.*2***
*ICR*	0.001***	0.002***
	(0.0001)	(0.0005)
*ICR* ^*2*^	-0.000003***	-0.000008***
	(0.000001)	(0.000002)
Constant	-0.332***	-1.022***
	(0.039)	(0.180)
N	4,050	4,050

Standard errors are in parentheses

*** p < 0.01

** p < 0.05

* p < 0.1. N/A indicates that the variables are omitted from the model.

## 5. Concluding remarks and implications

In 2021, when the Covid-19 pandemic began, Vietnam had a record high number of enterprises that temporarily suspended business activities, as significant market risk was the most severe at that time. Empirical studies have been conducted on either financial distress prediction models or market risk estimation during extreme events, but the current literature has largely overlooked the combined effects of these two important sources of risk for listed firms, particularly in Vietnam. As such, this study examines the effects of market risk and financial distress on the performance of 500 nonfinancial listed firms in Vietnam from 2012 to 2021. We employ dynamic panel models (the two-step difference- and system-GMM) a panel threshold regression in our empirical analysis. Market risk is estimated using CVaR, whereas financial distress is proxied by the ICR, the ratio of EBIT to interest expenses. As a result, the performance by listed firms in Vietnam is proxied by ROA and ROE.

Our empirical results indicate that a high level of market risk reduces firm performance, whereas a high ICR (i.e., low financial distress) improves firm performance. Market risk adds volatility to firms’ operations, resulting in lower performance. In addition, improving the ICR by increasing EBIT or decreasing interest expenses can improve firm performance. When the combined effects of market risk and financial distress on firm performance are considered, high ICR (i.e., low financial distress) can magnify the detrimental effect of market risk on firm performance, meaning that these negative effects can be heightened. Furthermore, our empirical results confirm the nonlinear (quadratic) relationship between financial distress and firm performance by listed firms in Vietnam. A high ICR, indicating low financial distress, is associated with better firm performance up to the threshold. After this threshold is exceeded, further increases in ICR will worsen firm performance. This finding indicates that an excessively high ICR indicates suboptimal use of leverage to improve firm returns. The optimal ICR for listed firms in Vietnam is between 112 and 134, depending on the firm size and industry, at which firm performance is maximized. An ICR level above 134 will reduce firm performance.

Based on the empirical results of our analysis, the Vietnamese government and the State Bank of Vietnam should coordinate in formulating and implementing monetary policy in line with other macroeconomic indicators, such as inflation and the business environment, to create conditions that will reduce interest rates for business loans. The government and the SBV should formulate appropriate policies to stabilize market interest rates.

Firms with low ICRs need to raise them to avoid financial distress. The firm’s executives should adjust pricing strategies periodically to simultaneously enhance revenue generation and cost management, thereby increasing EBIT to raise ICR. In addition, firm managers should maintain a reasonable debt level in the capital mix. Our empirical findings indicate that firms should maintain an ICR between 112 and 134 to optimize firm performance. Investors should consider ICR in their basic analysis to improve investment decisions.

The Vietnamese government needs to design and implement policies that support the stable development of the financial market. For example, investors trading in the Vietnamese market should be enabled to manage market risk by including different asset classes, such as fixed income, cash, and real estate, in their investment portfolios. Each of these asset classes reacts differently to an event that affects performance of the overall financial market in the Vietnamese context. In addition, investors in the Vietnamese financial market may need to use financial options, which are powerful tool for hedging their larger, diversified portfolios of stocks. At present, the Vietnamese financial market has only a limited number of products available. However, improvements are required to ensure that many products are available to meet different investors’ needs.

Our study exhibits limitations. Studies in the future may need to consider different proxies for market risk and financial distress to ensure the robustness of empirical findings. Our current study mainly focuses on the independent effects of market risk and financial distress on firm performance. Empirical studies in the future may need to consider the joint effects of market risk and financial distress on firm performance across sectors in Vietnam.
